# Treatment of the superior sagittal sinus and transverse sinus thrombosis associated with intracranial hemorrhage with the mechanical thrombectomy and thrombolytics: Case report

**DOI:** 10.1097/MD.0000000000009038

**Published:** 2017-12-08

**Authors:** Yuchun Liu, Keqin Li, Yi Huang, Jie Sun, Xiang Gao

**Affiliations:** Department of Neurosurgery, Ningbo First Hospital, Ningbo Hospital of Zhejiang University, Ningbo, Zhejiang, China.

**Keywords:** cerebral venous sinus thrombosis, chanical thrombectomy, morrhagic, rombolytic

## Abstract

**Rationale:**

The superior sagittal sinus (SSS) and transverse sinus are the major dural sinuses that receive a considerable amount of venous drainage. The occlusion of them has been suggested to cause intracranial hypertension, hemorrhage, and lead to potentially fatal consequences.

**Patient concerns:**

We reported a 35-year-old woman with headache presented to our emergency department with a decreased level of consciousness and epileptic seizures. The examination of speech, higher mental function, and cranial nerve were normal. Computed tomography (CT) demonstrated both subarachnoid and intraparenchymal hemorrhage and brain edema at the right temporal lobe accompanied by high density shadow in the right transverse sinus. Digital subtraction angiography (DSA) revealed extensive thrombosis of the SSS and bilateral transverse sinus.

**Diagnoses:**

The SSS and transverse sinus thrombosis, accompanied by right temporal lobe hemorrhage, subarachnoid hemorrhage (SAH).

**Interventions:**

An emergent mechanical thrombectomy by placed Solitair AB stent in the SSS, respectively, passed left and right sigmoid sinus–transverse sinus route. We removed the most clots, DSA revealed recanalization of the SSS and left transverse sinus was seen with normalization of the venous outflow, the occlusion of right transverse sinus was still present. There were 4 hours after patient back to neurosurgical intensive care unit (NICU), patient appeared anisocoria (R/L:4.0/2.5 mm), bilateral light reflexes disappeared, then we took a CT reexamination revealed intraparenchymal hemorrhage increased, brain edema was aggravated at the left temporal lobe, and mild midline shift. Subsequently, we performed decompressive hemicraniectomy and puncture the hematoma supplemented by B ultrasonic. Anticoagulation treatment was initiated 24 hours after surgery, and follow-up DSA showed gradually improved patency in the SSS and bilateral transverse sinus.

**Outcomes:**

Despite occlusion of the SSS and bilateral transverse sinus, patient's symptoms resolved after the operations and he was discharged without complications.

**Lessons:**

The favorable clinical outcome after complete occlusion of the SSS and transverse sinus, accompanied by right temporal lobe hemorrhage, SAH has rarely been reported and it might be explained by our timely surgical intervention and development of compensatory cerebral collateral circulation.

## 1 Introduction

Cerebral venous thrombosis (CVT) is a rare form of stroke, constituting 0.5% to 1.0% of all cerebral strokes,^[1]^ with an incidence of 13 to 16 per million annually.^[2,3]^ CVT is now being diagnosed with higher frequency due to increased awareness and ready access to magnetic resonance (MR) in developed countries. Multiple factors have been associated with CVT, but only some of them are reversible. Including surgery, prior medical conditions (e.g., thrombophilias, antiphospholid syndrome, cancer, inflammatory bowel disease), transient situations (e.g., pregnancy, puerperium, dehydration, infection), selected medications (e.g., oral contraceptives, exogenous hormones, substance abuse), and unpredictable events (e.g., head trauma) are some predisposing conditions.^[4–6]^ CVT may present with signs and symptoms of intracranial hypertension (headache, visual disturbances, papilledema, focal neurological deficits, and/or seizures).^[7]^ The mortality of CVT has decreased over the last few decades and is currently 5% to 10%^[8]^ and this is because of early recognition and treatment with anticoagulation.^[9]^ Currently, patient, who once CVT has been confirmed, should be initiated the treatment with anticoagulation even in the setting of intracranial hemorrhage (ICH).^[6]^ And the value of additional treatment such as systemic thrombolysis or endovascular treatment is currently being investigated.^[10,11]^ Besides, if neurological deterioration due to ICH or aggravate and cerebral edema, despite anticoagulation for CVT, decompressive hemicraniectomy should be considered.^[6]^ We present a case of successful mechanical thrombectomy, combined with the (local) use of thrombolytics, in a sober patient with an ICH due to CVT.

## 2 Case report

A 35-year-old woman with a history of headache for which she was taking Saridon, presented to our emergency department with a decreased level of consciousness and epileptic seizures. Initial symptoms had started 5 days earlier with severe headache more than before. She accompanied vomiting and nausea, no disturbance of vision, speech, or sphincter functions. On examination of speech, higher mental function, and cranial nerve were normal. There was no family history of venous or arterial thrombosis. She has experienced “laparoscopic hysteromyoma resection” at external hospital half a month ago, and she has been taking norethindrone.

**Figure 1:**
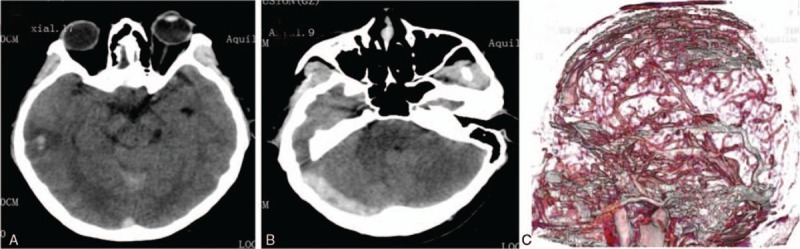
The results of 320CT before operation. A. Noncontrast CT head scan showed both subarachnoid and intraparenchymal hemorrhage and brain edema at the right temporal lobe; B. Noncontrast computed tomography head scan showed spontaneous hyperdensity of right transverse sinus; C. CT venography showed a contrast filling defect in the superior sagittal sinus and right transverse sinus. CT = computed tomography.

**Figure 2:**
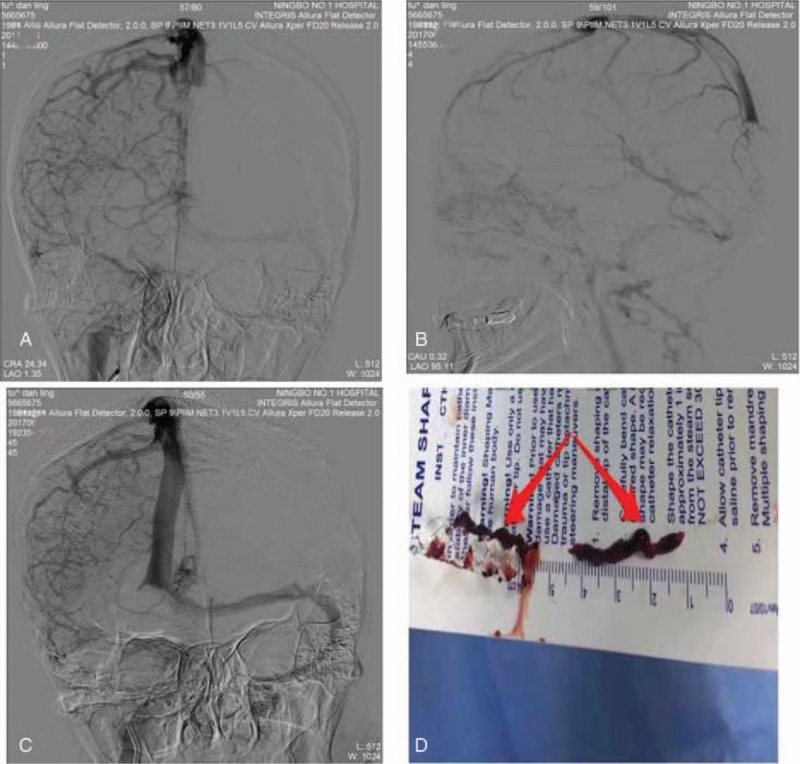
The results of mechanical thrombectomy and thrombolytics. A. Bilateral transverse sinus, and the right was more serious; B. On lateral digital subtraction angiography, the patient had a thrombus in the posterior SSS; C. The recanalization of the SSS and left transverse sinus was seen with normalization of the venous outflow, the occlusion of right transverse sinus was still present; D. The thrombus was entangled in the Solitaire AB. SSS = superior sagittal sinus.

Noncontrast CT showed both subarachnoid and intraparenchymal hemorrhage and brain edema at the right temporal lobe (Fig. 1A), accompanied by high density shadow in the right transverse sinus (Fig. 1B). Computed tomographic angiography (CTA) revealed a contrast filling defect in the superior sagittal sinus and right transverse sinus, indicating CVT (Fig. 1C). Informed consent was obtained. DSA revealed extensive thrombosis of the superior sagittal sinus (SSS) (Fig. 2A) and bilateral transverse sinus (Fig. 2B). We attempted to do mechanical thrombectomy by placed Solitair AB stent in the SSS, respectively, passed left and right sigmoid sinus—transverse sinus route. We removed the most clots (Fig. 2D), DSA revealed recanalization of the SSS and left transverse sinus was seen with normalization of the venous outflow, the occlusion of right transverse sinus was still present. Then, we placed a microcatheter into right transverse sinus and injected 500 × 10^3^ IU of urokinase by micro pump in 30 minutes. However, it did not has an obvious effect (Fig. 2C).

**Figure 3:**
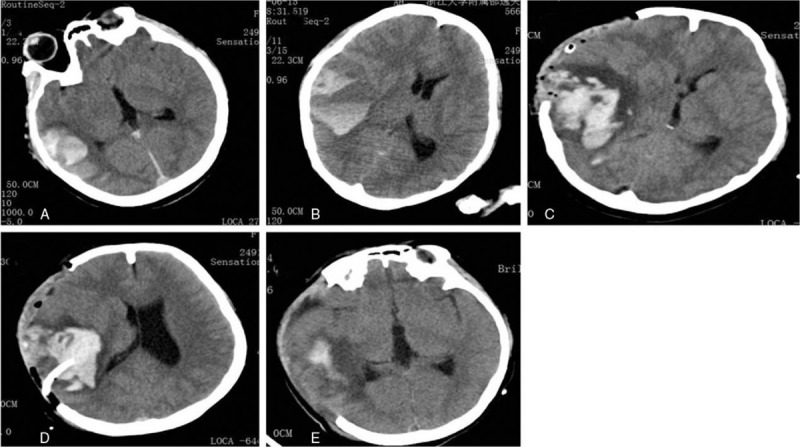
The results of all CT after mechanical thrombectomy and thrombolytics. A. Taken a CT reexamination at once after the endovascular therapy; B. Four hours after endovascular therapy; C. Two hours after decompressive hemicraniectomy; D. Two hours after the operation of punctured the hematoma supplemented by B ultrasonic; E. Two weeks after the endovascular therapy. CT = computed tomography.

**Figure 4:**
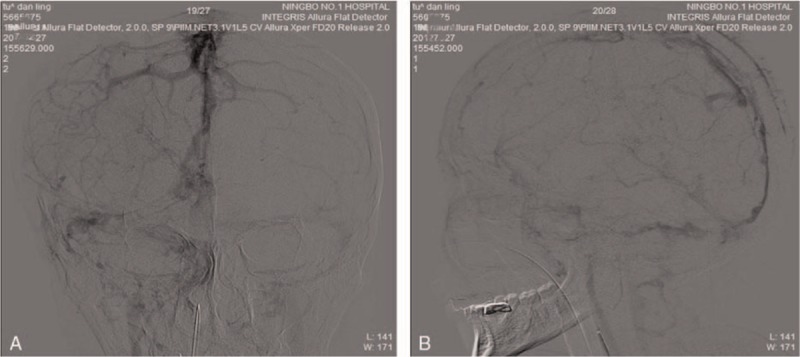
The results of DSA of 2 weeks after endovascular treatment. A. Posteroanterior radiograph; B. Lateral imagine. DSA = digital subtraction angiography.

**Figure 5:**
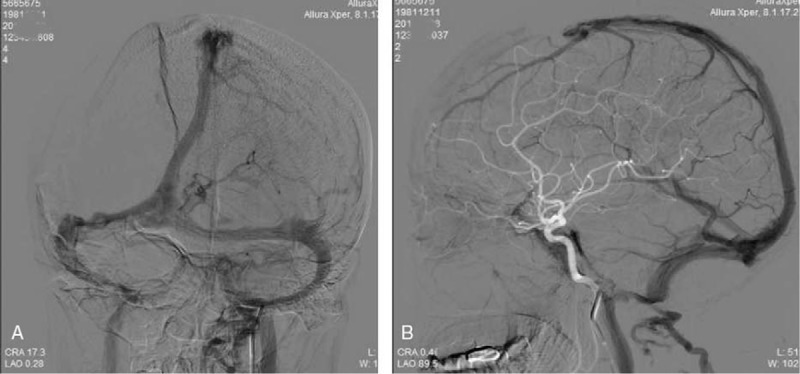
The results of DSA of 3 months after endovascular treatment. A. Posteroanterior radiograph; B. Lateral imagine. DSA = digital subtraction angiography.

After the intervention overed, we retook a CT scan showed increased intraparenchymal haemorrhage, but no midline shift (Fig. 3A). There were 4 hours after patient back to neurosurgical intensive care unit (NICU), patient appeared anisocoria (R/L: 4.0/2.5 mm), bilateral light reflexes disappeared. Then we took a CT reexamination revealed intraparenchymal hemorrhage increased, brain edema was aggravated at the left temporal lobe, and mild midline shift (Fig. 3B). Subsequently, we decided to select decompressive hemicraniectomy. After 2 hours of the operation, we taken a CT reexamination showed intraparenchymal hemorrhage increased more, brain edema was progressing (Fig. 3C). We attempted to puncture the hematoma supplemented by B ultrasonic. After 2 hours of patient backed to NICU, we taken a CT reexamination again and showed intraparenchymal hemorrhage reduced, lateral ventricles pressed was alleviate (Fig. 3D). Twenty-four hours after decompressive hemicraniectomy, neurological examination at presentation showed a Glasgow Coma scale of E4M6V5, isocoria, left-sided hemiparesis, and treatment with low molecular weight heparin (LMWH) in therapeutic dose was started. After 2 weeks we switched LMWH to oral anticoagulant therapy (vitamin K antagonist), and we taken a CT reexamination showed the intracranial hematoma was obvious decreased and the brain edema was alleviated (Fig. 3E). We performed a DSA revealed a good result of the recanalization (Fig. 4). Twenty days after mechanical thrombectomy, our patient was discharged and transfered to a rehabilitation hospital in good clinical condition that with complete recovery of mental status and only mild paresis of the left body. Three months after endovascular treatment, our patient did not respond to any discomfort and can walk by herself, review DSA showed: superior sagittal sinus and bilateral transverse sinus clear (Fig. 5).

## 3 Discussion

Although anticoagulation therapy is the most widely accepted therapeutic option for cerebral venous sinus thrombosis (CVST),^[6]^ approximately 3% to 15% of patients die in the acute phase of the disorder,^[12]^ some researchers believe it may not be the best choice for all patients and each patient needs to be individualized based on their presentation.^[13]^ Therefore, there have been several reports about the endovascular therapy (EVT) such as pharmacological thrombolytic, balloon angioplasty, stenting, microsnare, rheolytic thrombectomy, penumbra aspiration system, and Fogarty Embolectomy Catheter Balloon.^[14–18]^ This case report shows favorable clinical outcome after successful mechanical thrombectomy by placed Solitair AB stent and thrombolysis in a CVST patient presenting with ICH and subarachnoid hemorrhage (SAH).

Currently, thrombolysis combined with mechanical thrombectomy may strengthen the thrombolytic effect of drugs. By using the machine, the blood clotting is disrupted and form a tunnel, thus increasing the contact area between thrombus and thrombolytic, strengthening the thrombolytic effect of urokinase. At least, we reduced the total number of thrombolytic drugs and the bad response of it. According to Li et al,^[19]^ they reported that 14% and 6.8% of 52 patients with CVST had a mild increase in the parenchymal hemorrhage and achieved aggravation of the symptoms after receiving a 100–1500 × 10^3^ IU of urokinase during the mechanical thrombectomy, respectively. Although the percentage is low, it is potentially harmful for CVST patient presenting with ICH and SAH. In our case, we only used 500 × 10^3^ IU of urokinase by micro pump in 30 minutes for thrombolysis. Although we removed the most clots, our case presented with ICH increased and brain edema was aggravated after the endovascular therapy. Therefore, our patient experienced with decompressive hemicraniectomy and puncture the hematoma supplemented by B ultrasonic.

On the one hand, our case report demonstrates the clinical dilemma of the timing of anticoagulation for CVST presenting with ICH and SAH, and after hemicraniectomy. American Heart Association guidelines state that patients with CVT initial anticoagulation with adjusted-dose unfractionated heparin (UFH) or weight-based LMWH in full anticoagulant doses is reasonable, followed by vitamin K antagonists, regardless of the presence of ICH,^[6]^ but there are no specific recommendations about the timing of anticoagulation. Furthermore, there are no specific guidelines about the timing of anticoagulation for CVT who have undergone decompressive hemicraniectomy. In our case, 24 hours after decompressive hemicraniectomy, full dose of LMWH was started, based this decision mainly on clinical and radiographic stabilization of the ICH. In our opinion, there is a continuous increase in the intravenous pressure, this would lead to the hemorrhage and brain edema rising from the capillary or venous rupture. Therefore, it is mandatory to achieve an early recanalization of the occluded cerebral venous sinus in patients. And we started on warfarin after approximately 14 days of initial anticoagulation with LMWH. On the other hand, we confused that the limit of time of mechanical thrombectomy by stent with CVST. Guang-zhong et al^[20]^ found that the stent may injure the endothelium of vessel during the using of it and then caused new thrombosis, because there are reticular structure and arachnoid granules in the cerebral venous sinus.

In conclusion, anticoagulation is usually recommended for CVT,^[6]^ and was initially successful, our case proved that Solitaire AB combined with the (local) use of thrombolytics may be an alternative treatment option in patients with severe CVST for achieving revascularization both rapidly and efficiently available. Besides, it is important that there is an optimal medical management for patients with CVTS, and perform decompressive hemicraniectomy in time, when the patients with neurological deterioration due to severe mass effect or ICH causing intractable intracranial hypertension.
